# The assessment of circulating volume using inferior vena cava collapse index and carotid Doppler velocity time integral in healthy volunteers: a pilot study

**DOI:** 10.1186/s13049-016-0298-0

**Published:** 2016-09-02

**Authors:** Tom Peachey, Andrew Tang, Elinor C. Baker, Jason Pott, Yonathan Freund, Tim Harris

**Affiliations:** 1 Emergency Department, Royal London Hospital, Barts Health NHS Trust, Whitechapel Rd, London, E1 1BB UK; 2 Sorbonne université, UPMC univ Paris-06, Paris, France; 3 Emergency Department, Whipps Cross University Hospital, Barts Health NHS Trust, London, UK

**Keywords:** Inferior vena cava, Carotid Doppler, Passive leg raise, Circulating volume, Fluid resuscitation, Cardiac index

## Abstract

**Background:**

Assessment of circulating volume and the requirement for fluid replacement are fundamental to resuscitation but remain largely empirical. Passive leg raise (PLR) may determine fluid responders while avoiding potential fluid overload. We hypothesised that inferior vena cava collapse index (IVCCI) and carotid artery blood flow would change predictably in response to PLR, potentially providing a non-invasive tool to assess circulating volume and identifying fluid responsive patients.

**Methods:**

We conducted a prospective proof of concept pilot study on fasted healthy volunteers. One operator measured IVC diameter during quiet respiration and sniff, and carotid artery flow. Stroke volume (SV) was also measured using suprasternal Doppler. Our primary endpoint was change in IVCCI after PLR. We also studied changes in IVCCI after “sniff”, and correlation between carotid artery flow and SV.

**Results:**

Passive leg raise was associated with significant reduction in the mean inferior vena cava collapsibility index from 0.24 to 0.17 (*p* < 0.01). Mean stroke volume increased from 56.0 to 69.2 mL (*p* < 0.01). There was no significant change in common carotid artery blood flow. Changes in physiology consequent upon passive leg raise normalised rapidly.

**Discussion:**

Passive leg raise is associated with a decrease of IVCCI and increase in stroke volume. However, the wide range of values observed suggests that factors other than circulating volume predominate in determining the proportion of collapse with respiration.

**Conclusion:**

In contrast to other studies, we did not find that carotid blood flow increased with passive leg raise. Rapid normalisation of post-PLR physiology may account for this.

## Background

Accurate assessment of circulating volume is challenging in the acute setting. Pulse and blood pressure are neither sensitive nor specific in identifying hypovolaemia [1, 2]. Healthy adults may lose up to 30 % of their circulating volume with little change in their vital signs [2]. Furthermore, clinical parameters may be abnormal for reasons other than changes in circulating blood volume, such as tachycardia secondary to pain, or altered physiology due to medication [3]. Resuscitation with intravenous fluid aims to increase cardiac output, improving tissue oxygenation. Defining the volume and rate of fluid administration remains largely empirical, despite its fundamental importance in patient care.

Passive straight leg raise (PLR) increases venous return and effective circulating volume. PLR non-invasively delivers a fluid challenge of around 300 mL to the central circulation, allowing assessment of fluid responsiveness without fluid administration [4, 5]. An increase in cardiac output of >10–15 % in response to PLR predicts fluid responsiveness and this technique has been validated in spontaneously breathing patients so may be of use to emergency physicians [6, 7].

The inferior vena cava (IVC) may be measured by ultrasound. The proportion that the IVC collapses with respiration is termed the inferior vena cava collapse index (IVCCI = (IVC_max_ – IVC_min_)/IVC_max_). This has been shown to indicate fluid status in children [8], ventilated patients [9, 10] and healthy volunteers [11, 12]. Previous studies have demonstrated that IVCCI predicts fluid responsiveness in mechanically ventilated patients [9, 13] but data in spontaneously ventilating patients is less robust [14, 15]. At the time of this study, no study has evaluated IVCCI in relation to PLR.

Oesophageal Doppler is widely used in the intensive care environment to assess cardiac output and fluid responsiveness [16, 17]. Suprasternal Doppler measures blood flow through the aortic valve enabling non-invasive assessment of stroke volume and cardiac output (USCOM, USCOM Ltd., Sydney, Australia). Suprasternal Doppler has been validated against other measures of stroke volume in the emergency department, operating room and intensive care unit and shown to increase in response to PLR, [18, 19]. We therefore used this technique as a non-invasive reference standard for changes in stroke volume with a PLR in this study. Carotid arterial Doppler analysis is a simple, non-invasive method to assess circulatory flow. At the time of designing this study it had not been assessed as a surrogate measure of cardiac output or in response to PLR. Subsequently limited data has suggested it provides a good tool to assess fluid responsiveness [4, 20].

We hypothesised that IVCCI and carotid arterial flow would change predictably in response to a PLR, potentially providing non-invasive tools to assist clinicians in assessing the circulating volume and identifying fluid responsive patients. We tested this hypothesis by performing a pilot study on healthy volunteers after a period of no fluid intake.

## Methods

### Setting and participants

This prospective observational study was carried out in a non-clinical area within a UK Emergency Department. Ethical approval was granted by the National Research Ethics Service (NRES Ref: 13/LO/0472). Informed consent was obtained from all participants.

Healthy volunteers were recruited. The majority of participants were NHS students and medical or nursing staff. Inclusion criteria were: aged 18 years or over, able to straight leg raise and to comprehend written and spoken English. Exclusion criteria were pregnancy, body mass index > 40, atrial fibrillation, known ventricular dysfunction, valvular heart disease, hypertension, diabetes mellitus, chronic lung disease, inability to lie flat comfortably or have legs raised to 45°. Participants were fasted from food and drink for 8 h prior to scanning to maximise the probability of fluid responsiveness and as many ED attendees have had minimal fluid intake in the hours prior to arrival.

### Measurements and endpoints

**Fig. 1:**
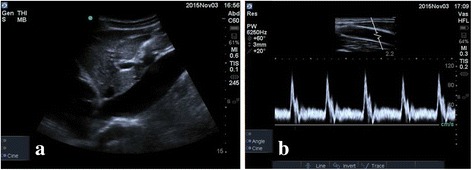
Example IVC and carotid artery ultrasound images. **a** IVC diameter measurements were taken 3-5 cm distal to the IVC-right atrial junction. **b** Common carotid artery diameter and Doppler flow were measured 2 cm proximal to the carotid bulb

Ultrasound measurements were carried out by one operator (TP) trained to College Emergency Medicine level-one standard. Additional vascular ultrasound training was obtained and measurements performed on 20 volunteers prior to commencing the study; images and technique were peer reviewed by an experienced operator (TH). A single operator was used as this was a proof of concept pilot study and we wished to exclude inter-observer variation. Ultrasound images were recorded using a SonoSite Edge (SonoSite Inc., Bothell, Washington, USA). IVC measurements were made with a curvilinear 3.5–5 MHz ultrasound transducer via the subcostal hepatic window just distal to the IVC-hepatic vein junction, approximately 3–5 cm distal to the right atrium, similar to previous studies [12]. The IVC was identified by phasic collapse with respiration, Doppler wave-form and compressibility (Fig. 1a). The right common carotid artery was identified by anatomy and wave-form characteristics. Carotid blood flow measurements were with a linear 7 MHz probe (Fig. 1b) [12].

Participants lay with legs flat and torso at 45^o^ for 5 min. Pulse rate was recorded from pulse oximetry blood pressure was recorded with an automated oscillometric sphygmomanometer, and respiratory rate was recorded manually. Ultrasound measurements in longitudinal B mode were made of the maximum and minimum diameter of the IVC occurring during 3 cycles of quiet respiration, and the smallest diameter occurring with a short sharp intake of breath (“sniff”) [12]. IVCCI was calculated as: (IVC_max_ – IVC_min_)/IVC_max_. A sniff index was calculated (IVC_sniff_) as: (IVC_max_ – IVC_sniffmin_)/IVC_max_. Ultrasound cardiac output monitoring was used to measure the blood flow volume across the aortic valve to calculate stroke volume [18]. Longitudinal scans of the right common carotid artery were taken in B mode and Doppler used to calculate the minute flow volume (the total blood flow through the right common carotid artery over 1 min) 2 cm proximal to the carotid bulb using a 5 mm angle adjusted Doppler gate. The participant’s torso was then lowered to horizontal and legs raised to 45 degrees. The measurements were taken immediately in the following order: suprasternal Doppler, IVCCI, then carotid flow and the participant returned to the initial position.

The primary endpoint was to assess the changes in IVCCI with usual respiration in response to PLR. Secondary endpoints were changes in the IVCCI with PLR using forced inspiration (‘sniff’); changes in the carotid artery blood flow with PLR; and correlation between carotid blood flow with cardiac output and carotid velocity time integral (VTI) with stroke volume. We measured the changes in cardiac output with PLR for reference.

### Analysis

All Gaussian distributed variables are expressed as mean and standard deviation (SD), and non-normally distributed variables were analysed as paired data and expressed as median and interquartile range (IQR). Means drawn from normally distributed data were compared with paired Student T test and non-parametric data were analysed with the paired Wilcoxon rank. Normality was assessed using Kolgomorov-Smirnov test. Correlation between stroke volume with carotid VTI and cardiac output with carotid flow was sought using Pearson test. A *p*-value of <0.05 was required for statistical significance, and all analyses were two-tailed. Data were analysed using SPSS (IBM SPSS Statistics 21.0, Chicago, Il, USA). No power calculation was possible for IVCCI and carotid Doppler changes with PLR as no previous work was available to base this upon, however, we estimated that a sample size of >30 subjects may be sufficient.

## Results

Thirty-four participants were recruited into the study. Data for one participant was not recorded correctly and was excluded from analysis leaving 33 (17 male, 16 female) data sets. The physiological changes observed in this study are summarised in Table 1.

**Table 1:** Physiological changes with PLR

Measurement/Indicator	Mean ± (SD) legs flat	Mean ± (SD) legs raised	% change of mean with PLR	Mean difference	*p* value
Pulse rate	61 ± (10)	60 ± (9)	-1.6 %	-1 (95 % CI -3 : +1)	>0.05
Respiratory rate	15 ± (2)	15 ± (2)	0.0 %	0 (95 % CI -1 : +1)	>0.05
Systolic blood pressure	115 ± (11)	107 ± (10)	-7.0 %	-8 (95 % CI -11 : -5)	>0.05
Diastolic blood pressure	72 ± (8)	65 ± (8)	-9.7 %	-7 (95 % CI -10 : -4)	>0.05
IVC-CI - quiet respiration	0.24 ± (0.12)	0.17 ± (0.10)	-29.2 %	-0.06 (95 % CI -0.10; -0.02)	<0.01
IVC-CI - sniff	0.57 ± (0.23)	0.42 ± (0.26)	-26.3 %	-0.15 (95 % CI -0.25; -0.06)	<0.01
Stroke volume (mL)	56.0 ± (13.0)	69.2 ± (14.2)	+23.6 %	+13.2 (95 % CI +9.6 : +16.7)	<0.01
Cardiac output (L/min)	3.3 ± (0.80)	4.1 ± (0.88)	+22.3 %	+0.75 (95 % CI +0.55; +0.96)	<0.001
Common carotid flow (mL/min)	770 ± (202)	773 ± (167)	+0.3 %	+3 (95 % CI -37 : +43)	>0.05

Changes in values of pulse rate, respiratory rate, blood pressure, cardiac output, IVCCI and carotid blood flow with PLR

PLR was associated with significant reduction in mean inferior vena cava collapsibility index from 0.24 to 0.17 (*p* < 0.01) – mean difference of -0.06 (95 % CI -0.10; -0.02). PLR was associated with a 23.6 % increase in mean stroke volume from 56.0 to 69.2 mL (*p* < 0.01), 18.2 % increase in cardiac output from 3.3 to 4.1 L/min (*p* < 0.001) – with a mean difference of 0.75 L/min (95 % CI 0.55; 0.96). Mean sniff collapsibility index reduced significantly from 0.57 to 0.42 (*p* < 0.01) – with a mean difference of -0.15 (95 % CI -0.25; -0.06). There was no significant change in carotid blood flow, from 770 mL/min to 773 mL/min (*p* = 0.8).

**Fig. 2:**
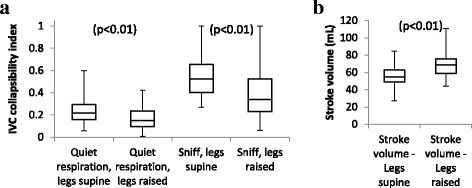
Respiratory changes of IVC collapsibility and changes of stroke volume on passive leg raise. (**a**) Box plot illustrating change in IVC collapsibility index for quiet respiration and sniff on passive leg raise. (**b**) Box plot illustrating change in stroke volume on passive leg raise

Results of the IVC collapsibility index are summarised in Fig. 2a. The IVC collapsibility index decreases on passive leg raise. One participant fully collapsed their IVC with a sniff with legs supine or raised.

Figure 2b shows PLR causes a significant rise in stroke volume from a median of 55 mL (IQR 49–63) to 69 mL (IQR 59–76). This demonstrates that stroke volume increases with passive leg raise as a consequence of increased central volume loading.

**Fig. 3:**
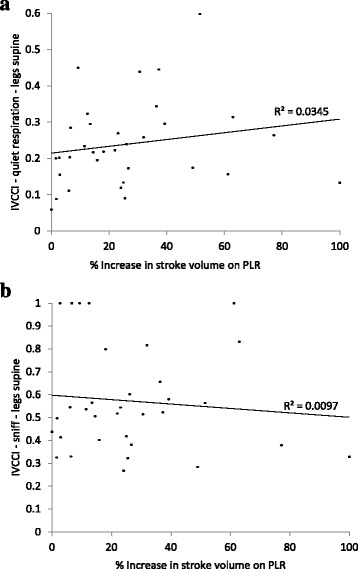
Correlations between IVCCI with the change in stroke volume on passive leg raise. (**a**) Correlation between IVCCI with quiet respiration and increase in stroke volume on passive leg raise. (**b**) Correlation between IVCCI with a sniff and increase in stroke volume on passive leg raise

Figure 3 shows the correlation between IVCCI with legs supine, both with quiet respiration and sniff, and % change in stroke volume with PLR. The data shows that although IVCCI reduces and stroke volume increases with passive leg raise (corresponding to the central fluid loading), there is poor correlation between IVCCI and stroke volume changes – IVC collapsibility does not predict the subjects in which fluid redistribution improves stroke volume.

**Fig. 4:**
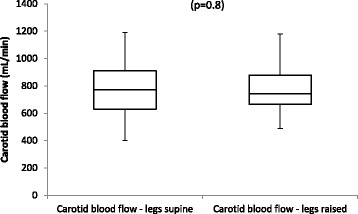
Changes in carotid blood flow with passive leg raise. Box plot representing changes in carotid blood flow with passive leg raise

Passive leg raise showed no significant alteration in carotid artery blood flow rate in our volunteers (Fig. 4).

**Fig. 5:**
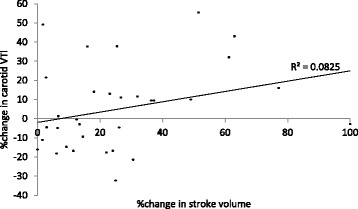
Correlation between change in carotid VTI with PLR and increase in stroke volume on passive leg raise

The change in stroke volume on PLR was plotted against the change in carotid VTI with passive leg raise (Fig. 5). This shows poor correlation between changes in stroke volume with changes in common carotid flow. Poor correlation was also observed when absolute values of stroke volume and carotid VTI with legs supine (*r*^2^ = 0.21) or raised (*r*^2^ = 0.29) were compared.

## Discussion

This study found that PLR was associated with a fall in IVCCI, increase in stroke volume and cardiac output, but no changes in carotid blood flow.

The magnitudes of IVCCI were greater with ‘sniff’ compared to quiet respiration, presumably due to greater changes in intrathoracic pressure. Mean reduction of IVCCI with PLR was similar with quiet respiration (29.2 %) and sniff (26.3 %) as a proportion of initial IVCCI.

We have shown that IVCCI, both with quiet respiration and ‘sniff’, decreases with PLR, suggesting that IVCCI alters with centralised blood volume and so may have potential to predict fluid responsiveness in spontaneously ventilating patients. However, we also found that the correlation between IVCCI and cardiac output was poor. The effect of PLR on IVCCI showed wide variation: ranging from a 96.6 % reduction in quiet respiration IVCCI with PLR and a 92.4 % reduction in sniff IVCCI with PLR to a decrease in IVCCI on PLR: opposite to the expected finding. Eight participants had a greater IVCCI post-PLR with quiet inspiration and five post-PLR with ‘sniff’ inspiration. These data suggest that variations in inspiratory force and volume may have a greater effect on IVCCI (and cardiac output) than volume centralisation with PLR in some subjects. The position change may affect respiratory effort and mechanics. Variability of IVCCI with breathing method has been reported by Kimura, who noted for the same inspiratory tidal volume diaphragmatic breathing caused greater IVCCI than chest wall breathing [21]. Thus IVCCI may have limited clinical potential unless inspiratory effort is standardised both between measurements and participants. Future work exploring the role of IVCCI should attempt to standardise respiratory effort.

Since our study was performed others have also explored the role of IVCCI and PLR. Corl [22] examined changes in IVCCI with PLR in spontaneously ventilating patients presenting to an adult emergency department, finding a mean IVCCI of 15.8 %, with IVCCI 4 min after PLR decreasing by an average of 0.5 %. The authors concluded IVCCI was a poor predictor of fluid responsiveness. This may also be consequent upon the 4-min delay between PLR and IVCCI measurement, as haemodynamic changes following PLR are transient as discussed further below. Panebianco [23] showed that IVC size and IVCCI are little changed when recorded supine or at 45 degrees, concluding that measurements taken supine and semi-upright may be regarded as equivalent. Lanspa examined IVCCI in 14 spontaneously ventilating ICU patients receiving 10 mL/kg intravenous fluid delivered over 20 min [24]. Fluid responsiveness was defined as increase in cardiac output of >15 %. Five subjects were fluid responders and nine were non-responders. An IVCCI of >15 % had a positive predictive value of 62 %, but excellent negative predictive value of 100 % (*p* = 0.03). Muller examined 40 spontaneously ventilating ICU patients with acute circulatory failure [25]. Muller found that IVCCI of >40 % had a positive predictive value of 72 % for fluid responsiveness (defined by increase in subaortic VTI of >15 % following infusion of 500 mL 6 % hydroxyethylstarch), but IVCCI of <40 % had a negative predictive value of 83 % and so could not exclude fluid responsiveness. Airapetian examined 59 spontaneously breathing ICU patients with acute circulatory failure [26]. This study found that an IVCCI of >42 % distinguished between fluid responders (>10 % increase in cardiac output with 500 mL 0.9 % saline infusion) and non-responders with high specificity (97 %) and positive predictive value (90 %) but low sensitivity.

In our study the changes in cardiac output measured by USCOM were short-lived and values returned to their pre-PLR values within around 30 s to 2 min. A recent study concurs; reporting that PLR results in a transient change in physiological measurements, with normal physiology restored within one to 4 min [27]. The time taken to restore to normal physiology was not an a priori measured variable in our study, as this rapid normalisation of physiology was not anticipated. However, this was noted consistently in all subjects, anecdotally, more rapidly in fitter and more athletic subjects.

The time to maximum change in stroke volume and return to baseline with PLR is not well defined. A study of healthy spontaneously ventilating volunteers by Delerme [6], measured changes in pulse oximetry waveforms with PLR, finding a significant difference between baseline and post-PLR, which were maintained for 5 min. Boulain reported haemodynamic changes persisting for 4 min post-PLR in mechanically ventilated ICU patients [28]. However, Lamia [27] notes that haemodynamic changes following PLR are transient so designed their study to record their data within 1 min of PLR. Best practice for performing a passive leg raise is discussed by Monnet [29], who notes that physiological effects from PLR may vanish after 1 min. This paper outlines how PLR should be optimally performed to assess for changes in stroke volume and discusses how differences in results in the literature may be due to differences in methods of PLR. The short duration of the PLR associated changes in stroke volume were not known at the time of conducting this study and consequently the exact times of each component of the study were not recorded. This may explain the range of changes in cardiac output reported here. The limited time window during which measurements should be taken potentially limits the utility of PLR without continuous cardiac monitoring.

Mean stroke volume increased 23.6 % from 56.0 to 69.2 mL. Mean cardiac output increased by 22.3 % from 3.3 L/min to 4.1 L/min with PLR, comparable to other studies [6, 26, 27]. A rise in cardiac output >10 % with 500 mL crystalloid bolus or PLR is commonly used to define fluid responsive patients [9, 10]. In our study 24 participants increased their cardiac output by >10 % on PLR and were therefore fluid responsive. Nine participants had changes in cardiac output of <10 %. This is surprising as all participants were expected fluid responsive as they had normal cardiovascular systems, were well and fasted for 8 h. Review of the quality of the suprasternal Doppler traces identified them as comparable to previous work and do not account for the non-responders. This finding may reflect the transient effect on cardiac output consequent upon PLR. The USCOM cardiac output readings in this study are lower than may be expected for healthy volunteers. A related study on this machine performed by a different operator within our institution also obtained lower than expected cardiac index readings [12]. A meta-analysis by Chong and Peyton [30] comparing USCOM with thermodilution noted that USCOM underestimates cardiac output by an average of 0.39 L/min with large variation between these methods of cardiac output measurement.

Our study reports that common carotid artery flow does not increase in response to PLR and we found no relationship between carotid artery blood flow and stroke volume, irrespective of leg positioning. This suggests that carotid VTI cannot be used as a surrogate measure for changes in stroke volume on PLR in healthy volunteers. This is contrary to a recent study by Marik where carotid artery blood flow differentiated fluid responders from non-responders [4]. In their intensive care unit based study of 34 patients, 17 non-responders to fluid boluses had minimal changes (mean increase 0.1 %) to carotid blood flow on PLR, while 17 fluid responders had a mean carotid blood flow increase of 79 %. This paper notes that work in their group on healthy volunteers found PLR caused an average 33 % increase in stroke volume and 16 % increase in carotid blood flow. However, there are differences in study design.

The Marik study measured the diameter of the carotid artery and found that this changed with fluid loading. Our study did not measure the carotid artery diameter, assuming no change with PLR, and thus our results may represent a false negative finding. Marik studied patients with septic shock in intensive care who had been partially or completely fluid resuscitated. Carotid blood flow changes in response to PLR maybe different in patients with sepsis as compared to healthy volunteers, possibly as a consequence of altered autoregulation [31]. As discussed, the changes in stroke volume consequent upon PLR are short lived and the carotid artery measurements were made at the end of our protocol, so delay in obtaining readings may result in recordings already returning to baseline. Gassner [20] also investigated the role of carotid artery ultrasound as a surrogate marker for cardiac output Their study on ICU patients found good correlation between carotid artery flow when compared with PA catheter (intra-class correlation 0.74) or arterial waveform pulse contour analysis (intra-class correlation 0.84) over a wide range of cardiac outputs.

### Limitations

There are several limitations to this study. The study measurements were all taken by one operator (TP). Our data consequently allows no comment on inter-observer reliability. However, this was a pilot study designed to test our hypothesis in healthy volunteers and not the clinical utility of IVCCI or carotid blood flow with PLR. Secondly, this pilot proof of concept study was performed using healthy volunteers and not patients. Therefore, the results are not transferable to the clinical environment. We aimed to use this work to inform future clinical studies.

IVCCI is dependent on factors other than circulating volume. The rate and volume of inspiration, pulmonary pathology and cardiac disease all impact the intrathoracic pressure. Studies on mechanically ventilated patients standardise tidal volume by ideal body weight [9, 10]. We believe that future studies exploring the role of IVCCI should focus on controlling and standardising these parameters if IVCCI is to find a role in spontaneously ventilating patients.

In our study we found that changes in physiology following PLR were transient and rapidly normalise, often returning to baseline values in around 1 min following PLR. Obtaining readings for IVCCI, carotid blood flow and cardiac output within this time frame is challenging using the non-invasive technology evaluated in this study. The time required all data acquisition was not formally recorded and it is likely that some values were obtained after this time period, while the physiological responses to PLR were diminishing.

## Conclusions

This study reports an increase in stroke volume and fall in IVCCI with PLR in healthy volunteers. The wide range of IVCCIs reported with PLR suggests that the technique requires development prior to clinical use. In contrast to previous studies we report that common carotid artery blood flow does not change with PLR. The haemodynamic changes on the healthy volunteers following PLR were transient and rapidly returned to the baseline values, which may account for these findings. This may limit the role of PLR as an indicator of fluid responsiveness when using non-invasive monitors unable to provide continuous readings.

## Abbreviations

CI: Confidence interval
IQR: Interquartile range
IVC: Inferior vena cava
IVCCI: Inferior vena cava collapse index
PA: Pulmonary artery
PLR: Passive leg raise
SD: Standard deviation
SV: Stroke volume
VTI: Velocity-time integral
